# Dr. E. J. Wolff

**Published:** 1916-12

**Authors:** 


**Dr. E. J. Wolff** (not listed in State Directory), died Nov.

20 in E. Aurora, aged 25. He had recently come from Chicago.

